# Perceptions, Motivations, and Empowerment Strategies of Midwives in Rural and Remote Areas of Northern Morocco

**DOI:** 10.3390/ijerph192214992

**Published:** 2022-11-14

**Authors:** Abdelouahid Louazi, Antonio Frías-Osuna, Catalina López-Martínez, Sara Moreno-Cámara

**Affiliations:** 1 Higher Institute of Nursing Professions and Health Techniques of Tetouan (ISPITST), Tetouan 93020, Morocco; 2 Department of Nursing, School of Health Sciences, University of Jaén, 23071 Jaén, Spain

**Keywords:** nurse midwives, rural health services, motivation, empowerment, qualitative research

## Abstract

The shortage of midwives is a problem in rural and remote areas. This is mainly the consequence of job insecurity and difficult living conditions. The present study aimed to identify and analyse the perceptions and motivations of midwives in rural and remote areas of northern Morocco on the quality of their working life and the motivational factors and empowerment strategies they use to maintain and develop their work. It is a qualitative study that follows Van Manen’s hermeneutic phenomenology approach. Three focus groups and in-depth interviews were conducted with 15 midwives from rural and remote areas. The results indicate that midwives in rural and remote areas have a negative perception of the quality of the work and their personal life because of the scarcity of basic resources, unfavourable working conditions, and the personal sacrifices they have to make to support themselves. However, some factors favour their efforts. Therefore, there is a need to promote intersectoral policies that focus on improving material and human resources, as well as the working and personal conditions of midwives and the factors that support and empower them.

## 1. Introduction

Many women, children, and adolescents have little or no access to good-quality essential health services [1]. This has an impact on their annual mortality rate, which remains unacceptably high, according to the World Health Organization (WHO), and stood at 290,000 maternal deaths in 2017. These were the result of complications in pregnancy and childbirth [2]. The WHO estimates that 2.8 million pregnant women and newborns die each year (at a rate of 1 every 11 s) [2,3]. Many of these deaths could be prevented with appropriate health and social policies, according to the WHO and the United Nations (UN) [1,4].

In order to improve these figures, numerous strategies have been put in place at the international level in line with the Sustainable Development Goals (SDGs) [5,6]. The updated Global Strategy for Women’s, Children’s, and Adolescents’ Health (2016–2030) [1] aims to accelerate the momentum for women’s, children’s, and adolescents’ health by strengthening the availability of a sufficient number of skilled and well-equipped health workers, ensuring good quality services with innovative approaches, and improving monitoring and evaluation [1,7].

At the local level, most countries that have signed up to the goals of the Global Strategy and the SDGs have rapidly increased coverage of care for women, children, and adolescents, but with significant equity gaps among the poorest groups [8,9,10]. However, according to the 2019 Sustainable Development Goals Report [4], progress has slowed in recent years. In Morocco, the 2018 National Population and Family Health Survey [11] showed a 35% decrease in maternal mortality between 2010 and 2016 and an average annual reduction rate of 7%. The same downward trend was observed for neonatal mortality, from 21.7 to 13.56 per 1000 live births [11]. However, wide inequalities in access to obstetric care persist between urban and rural areas and socio-economic levels [12]. The maternal mortality ratio in rural areas is twice as high as in urban areas (148 versus 73 per 100,000 live births) and the proportion of deliveries in a supervised setting does not exceed 55% for rural women [13]. The availability, distribution, and skills of human resources is another major constraint, especially in rural and remote areas [14]. The objectives of the Action Plan to Accelerate the Reduction of Maternal and Infant Mortality in Morocco [15], as well as the National Strategic Plan for Reproductive Health, which are currently being implemented, seek to strengthen and reorganise existing facilities and services. The aim is to ensure maximum synergy and coverage, as well as to enhance the development of the competencies of health professionals caring for women during pregnancy, childbirth, and the postpartum period [14].

Health care for half of the world’s population that live in remote or rural areas (primarily in lower-middle-income countries, such as Morocco) is made extremely difficult by the fact that the number of qualified and motivated health professionals is low; only 38% of nurses and 24% of all medical staff work in these regions [16,17]. This is related to the internal and international migration of health workers dissatisfied with living and working conditions in rural areas. It explains why the coverage of qualified midwives is least equitable between rural and urban settings [8,12,18]. To encourage health professionals to stay in rural areas, the WHO has made recommendations organised in five blocks (training, financial incentives, personal, professional, and regulatory support) that can be adapted to the realities of each setting [16].

The factors that influence health professionals’ decisions to move to, stay in, or leave remote or rural areas are highly complex. They encompass personal characteristics, the structure of the health system, and the social, economic, and political environment [19]. In turn, the influence of background motivation, be it economic, social, cultural, religious, or otherwise, is strong [20]. One of the most widely accepted models used to explain the factors that influence the attraction and retention of professionals in rural areas of low- and middle-income countries is that proposed by Lehmann et al. [21]. It consists of five dimensions: (1) the international environment (salary, working conditions, and opportunities); (2) the national environment (e.g., political and social stability and the condition of public services); (3) the local environment (general living conditions such as housing, transport, schools, sanitation, electricity, as well as the degree of social isolation); (4) occupational factors (management of health professionals, professional relationships, professional development opportunities, infrastructure, equipment, and job satisfaction); and (5) individual factors (i.e., socio-demographic characteristics) [21]. Each affects the capacity of health professionals to make autonomous decisions and to have a greater sense of control and influence over their situation [22].

Several studies have been conducted on the motivation and empowerment of healthcare professionals in different cultural contexts [23,24,25], in urban areas, and in high-income countries [26,27]. They have primarily employed quantitative methodologies [28,29], and, therefore, do not provide much information about human decisions and motivations. The present study explores the experience of midwives working in rural and remote areas of Morocco using a qualitative approach.

The study aims to explore the perceptions and motivations of midwives in remote and rural areas of northern Morocco about the quality of their working life and the empowerment strategies they use to maintain and develop their work in such an environment.

## 2. Materials and Methods

### 2.1. Design

A qualitative study was carried out with a hermeneutic phenomenological orientation based on Van Manen’s theories [30]. The philosophical aim of phenomenology is to provide an understanding of the participant’s lived experiences. This approach was considered suitable for exploring and understanding the experiences of midwives practising in rural areas, including their perceptions and the meanings attributed to facts and events in their practice [31].

### 2.2. Participants

The participants comprised 15 female midwives (Table 1 and Table 2) who had been providing services in community health and delivery centres in rural communities in hard-to-reach areas of the Tangier-Tetouan-Al Hoceima region of north Morocco for 1 year. The single-sex nature of the sample mirrored the profile of the profession in the area. The participants were selected by purposive sampling of maximum variation [32] so that the richest possible occupational experience could be obtained. Potential participants were identified using the personnel registers kept by the human resources service of the Regional Health Directorate of Tangier-Tetouan-Al Hoceima. The participants were contacted directly and, after confirming that they met the criteria for inclusion and having had the objectives of the study explained to them, agreed to take part. They then filled in an informed consent form.

### 2.3. Data Collection Methods

Fifteen semi-structured in-depth interviews and three focus groups were convened, thus triangulating the data collection methods. This made it possible to approach the study in different ways and to make the study more valid, reliable, and precise. The internal consistency of the results could also be more accurately gauged [33]. The study was conducted in community health and birth centres in three different provinces. The data were collected between April 2018 and December 2019.

Both the interviews and the focus groups were led by the lead researcher. The interview script used was previously agreed upon with the research team. It covered perceptions of the work environment, motivations and factors in the participants’ decision to stay in rural and remote areas, and individual/collective empowerment strategies. The interviews and the focus group were audio-recorded, transcribed, and analysed while taking into account the notes made in the field notebook.

### 2.4. Ethical Considerations

The study had the approval of the Jaén Ethical Research Committee and the Ethics Committee of the University of Jaén. Ethical standards were abided by at all stages in accordance with the bioethical principles of the Declaration of Helsinki [34]. As was noted above, the participants gave their consent to involvement. They were provided with information relating to the study in verbal and written form. Confidentiality and anonymity were guaranteed, so each participant was given a pseudonymous name; all identifying information was deleted.

### 2.5. Analysis

The analysis was carried out according to a hermeneutic phenomenology approach suggested by the theories of Van Manen [30] and aided by the methodology of Giorgi [35]. The first stage involved a review of the literature in which the theoretical assumptions were clarified. At the second stage, fieldwork was carried out, in which information was obtained about the participants’ lived experience. At the third stage, we proceeded to conduct the analysis. The transcripts of the interviews and focus groups were carefully read, then the units of meaning were extracted and grouped into codes. The codes with common elements were grouped into subcategories and categories with a higher level of meaning. Five core categories, ten subcategories, and nineteen codes emerged. During the coding process, and especially in the final stages, analysis was carried out not only at the syntactic level, but also at the pragmatic level, when each of the categories, subcategories, and codes were interpreted and integrated. These, along with their naming and meaning, were triangulated. The data analysis software package NVIVO 12 (QSR International, Victoria, Australia) was used to assist in the coding and analysis.

## 3. Results

**Figure 1:**
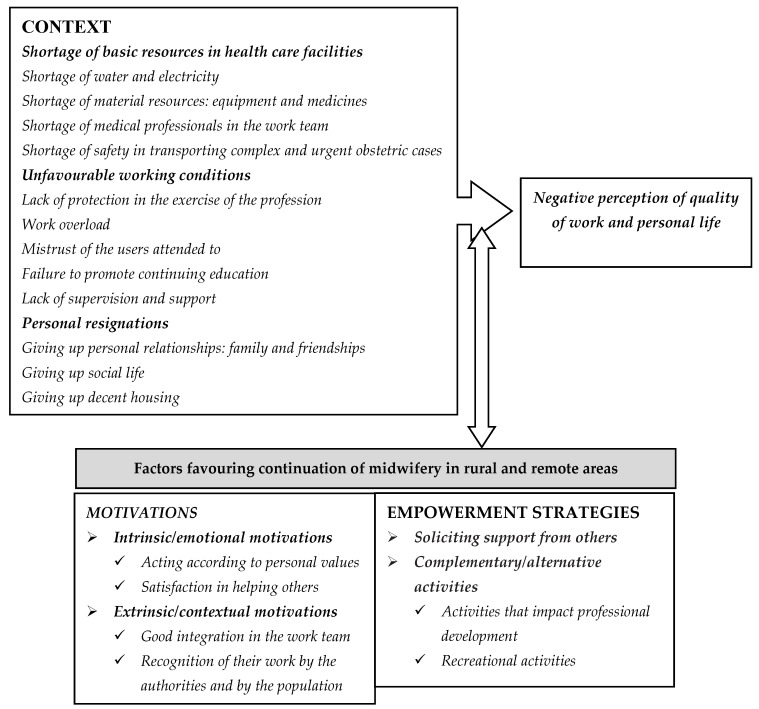
The model of perceptions, motivations, and empowerment strategies developed by midwives in rural areas of northern Morocco.

Midwives working in the rural and remote areas of northern Morocco work in an environment characterised by a shortage of basic resources in health care facilities and unfavourable working conditions. It is difficult for them to ensure quality obstetric care that guarantees the safety of either the pregnant woman, the newborn, or themselves. In addition, the personal sacrifices that midwives make to carry out their work in this context increase the desire to leave their jobs, while the lack of government measures to improve matters increases the negative perception of the quality of work and personal life. However, some factors motivate and empower them to continue their work in these hostile circumstances (Figure 1).

### 3.1. Shortage of Basic Resources in Health Care Facilities

The environment in which midwives work lacks the minimum conditions and materials to provide adequate care for pregnant women and newborns.

#### 3.1.1. Water and Electricity Shortages

The various health care centres in the remote and remote areas of northern Morocco where pregnant women are cared for lack basic resources, such as water and electricity, on a continuous basis, particularly at the time of delivery. This situation makes it difficult to attend births, especially at night, and affects the quality of care for postpartum women.

*“…the unavailability of drinking water in the health centre stands out… there are frequent electricity restrictions, especially in the summer…”*

Karima

*“…heating equipment is needed to combat the heat and cold…”*

Latifa

#### 3.1.2. Shortage of Material Resources: Equipment and Medicines

In some centres, basic equipment such as newborn warmers, ultrasound scanners, or ultrasound foetal heart rate monitors are missing or out of order. There is also a shortage of medicines necessary for the care of the mother and the newborn. This results in very limited care and complications (some of which are life-threatening), problems that could be resolved with the appropriate means.

*“Some essential drugs are needed in obstetrics, such as oxytocin and vitamin K.”*

Latifa

*“Warmers are needed for the babies…”*

Latifa

*“There are no warmers, medicines, oxygen therapy, neonatal resuscitation equipment…”*

Rachida

#### 3.1.3. Shortage of Medical Professionals in the Work Team

The shortage of gynaecologists in the rural and remote areas of northern Morocco leaves midwives without the corresponding medical support during childbirth and the postpartum period, which makes their task more difficult and increases risk for the midwives, especially in more complex cases. This makes them feel insecure and fearful.

*“…there is a great unavailability of gynaecologists at the Uezzan Hospital. At least four gynaecologists are needed to ensure on-call duty.”*

Laila

*“There is no medical support, only general practitioners who do not have obstetric skills attend the centre.”*

Ibtissam

#### 3.1.4. Lack of Safety in the Transport of Complex and Urgent Obstetric Cases

Due to the lack of adequate material and professional equipment in health care centres in rural and remote areas of northern Morocco, midwives refer the most complex obstetric cases to better- equipped hospital centres. They highlight the risks involved in making these referrals without having the competence to do so, along with the great distance and poor state of the roads.

*“…there is also the problem of transfers without a hospital admission order…, we run the risk of transferring the parturient to different hospital establishments without legal cover…at least 4 h between going and returning with a winding road in conditions that are uncomfortable for any kind of care and assistance…”*

Ikram

*“…there are climatic and geographical access difficulties: snow and blocked access, which causes difficulties in transferring cases…”*

Ibtissam

*“…transfers to the maternity hospital are unprotected due to the absence of a mission order…”*

Nada

### 3.2. Unfavourable Working Conditions

In the course of their professional activity, midwives have to deal with other factors related to working conditions that hinder their work and motivation.

#### 3.2.1. Lack of Protection in the Practice of the Profession

Midwives ask for more protection when they have to make home visits in urgent cases. They report that the homes are remote and in a deplorable state. Having to travel alone as women places them in a situation of considerable vulnerability. For these reasons, they have stopped delivering babies at home.

*“…We demand security, but there is none, we have stopped attending births now, because we do not have the minimum conditions for obstetric practice, such as safety at work…”*

Latifa

*“…Unavailability of a security officer at the health centre…”*

Hind

#### 3.2.2. Work Overload

The midwives emphasise that when they are required to work full-time on-call, they feel particularly stressed as they have no rest periods. The reason for this is that they feel that they never switch off, as in any emergency, the responsibility would fall on them.

*“…you feel more relaxed and less stressed when you do your work during normal working hours and once you start working on call, the stress starts, even if there is no workload…”*

Naziha

#### 3.2.3. Mistrust of the Clients Attended to

The midwives highlight the problems of communication and trust with the client population. The midwives perceive that their role is not recognised as it should be, which causes obstacles in communication and hinders their professional work. In addition, the different dialects and cultures of the different areas in these rural and remote areas of northern Morocco make it difficult to understand and implement the midwives’ recommendations.

*“…and communication with the population. Sometimes, when you speak and hint at something, the others interpret you in a different way…”*

Naziha

*“…aggressive and unsympathetic behaviour of the population.”*

Ibtissam

*“…very low literacy rate, which makes it difficult to convince women to go for specialised consultations…”*

Hind

#### 3.2.4. Non-Promotion of Continuing Training

The midwives feel that there is a lack of promotion and provision of health training programmes by the health institutions themselves. This makes them feel forgotten, as if they are being used to cover this most vulnerable population, but no one cares about them.

*“…There is no continuous training, for a period of one semester there has been no continuous training*.”

Naziha

*“…lack of continuous training and retraining of midwives…”*

Nada

#### 3.2.5. Lack of Supervision and Support

The midwives claim that there is a lack of supervision of health programmes and initiatives to support their work as midwives. This limits the development of their competencies and demotivates them in their professional practice.

*“There is also a lack of supervision of health programme managers. Since we started working here, we have never received visits from them to share with us news and updates on health programmes…”*

Naziha

*“…there is a lack of support…”*

Najoua

### 3.3. Personal Resignation

When midwives go to work in rural and remote areas, they assume that they will not have the amenities, family, or social life that they would have in urban areas. In addition, leisure and other opportunities for personal and professional growth are diminished by the location of their workplace. They feel that they live in a closed circuit characterised by isolation and monotony.

*“…you are finally inside the accommodation, if there is a delivery you attend it, if not, you stay in the same place, that is to say that you are always in a closed circuit. This situation is repeated every day.”*

Naziha

#### 3.3.1. Giving up Personal Relationships: Family and Friendships

In their work in rural and remote areas, midwives experience a physical separation from their family and friends, who are not only far away, but also difficult to reach. This leads to feelings of isolation and loneliness.

*“…socially, we live in isolation from our family and friends.”*

Hasna

*“We find it difficult to travel and visit family at weekends, which leads to isolation and loneliness…”*

Ibtissam

#### 3.3.2. Withdrawal from Social Life

The midwives are located in places where there are no spaces for leisure outside of nature, such as shopping centres, cafés, public establishments, and so on. Thus, they experience constant and frustrating monotony.

*“…you are finally inside the accommodation, if there is a birth you attend it, if not, you stay in the same place, i.e., you are always in a closed circuit. This situation is repeated every day.”*

Naziha

#### 3.3.3. Denial of Decent Housing

The housing provided to midwives when they work in rural and remote areas is small and shared with other workers. Electricity and drinking water are regularly not available. When there are no deliveries to attend, they spend most of their time in the house.

*“…the unavailability of drinking water in the house reserved for midwives on a permanent basis… there are frequent electricity restrictions, especially in the summer.”*

Karima

*“In addition, the housing provided to midwives working in these areas is shared and very narrow… There is only one place of accommodation… to serve as a residence where you have no space to move around…”*

Laila

### 3.4. Negative Perception of Quality of Work and Personal Life

Due to the above, the midwives feel constant dissatisfaction and frustration. They were aware that working in such conditions would be difficult, and they do not expect health institutions to become involved in improving them, nor do they feel that they can bring about any change. Moreover, despite all their efforts, they perceive a constant external demand to perform better. Their way of thinking is to adapt to the present circumstances and to trust in their ability to find solutions to each situation by improvising with the means at their disposal.

*“We cannot speak of quality in our work because we work in a rural context, in a remote area. There is no quality if not how you can arrange things according to the circumstances that arise.”*

Hanane

*“…performance incentive…”*

Najoua

Such unfavourable conditions, both in terms of work and personally and socially, mean that they aspire to leave their jobs. They live with the constant idea of escaping as soon as they have the opportunity and going somewhere more conducive. Some have considered the possibility of changing careers.

*“Frankly, I’m thinking of changing my profession altogether, continuing my university studies… I want to be close to my family.”*

Ikram

*“We are always waiting for staff to move to a new job…”*

Laila

### 3.5. Factors Favouring the Continuation of Midwives in Rural and Remote Areas

Despite the very hostile context, the midwives say that certain factors motivate them to stay in these areas and encourage them to empower themselves. However, when these are lacking, they express a desire to leave their jobs or even change profession.

#### 3.5.1. Intrinsic/Emotional Motivations

##### Acting According to Personal Values

At the same time, midwives are motivated to stay at their jobs by the responsibility that underlies the ethical, religious, deontological, and professional values that prevail in them. The “love of the profession” is also an incentive to motivate midwives to stay in the workplace.

*“I studied this to be able to help women, and in these places, we can do a lot for them…”*

Hanane

*“For me, my motivations for the development of patient care in rural areas are, first of all, the love of the profession because I have chosen a job that brings me closer to God.”*

Hanane

##### Satisfaction from Helping Others

Feeling part of actions that benefit the most disadvantaged gives the midwives great satisfaction and compensates, in part, for the sacrifices they are making. They are aware that people’s lives are in their hands and that they directly improve the quality of life of pregnant women and their families who thank them for preventing deaths. They also take comfort in being able to provide equitable and fair treatment, considering the inequality and vulnerability of the women they help.

*“…I voluntarily participate in associative health activities such as the organisation of medical caravans for rural populations in remote areas. These strategies strengthen me and offer me a respite from the effects of the tensions experienced at work…”*

Hanane

*“Assisting a woman in labour to give birth to a newborn is for me the most wonderful feeling of joy I can feel. It gives me strength and motivation to overcome the difficulties I encounter at work.”*

Zohra

#### 3.5.2. Extrinsic/Contextual Motivations

##### Effective Integration into the Working Team

Aspects such as having a close-knit, supportive, and cooperative working team in which each member is recognised as valid and important are considered by the midwives as an incentive to stay in their job, along with good interpersonal relations.

*“There are some positive factors such as… good understanding and respect among the team members…”*

Latifa

*“Regarding motivation at work, the thing that relieves me from the difficulties of the rural work context is that we have a united, understanding, and very cooperative work team.”*

Hasna

##### Recognition of Their Work by the Authorities and the Population

The recognition of their contribution by the local authorities and the generosity and gratitude of the population they serve incentivises them to remain in the role.

*“The good treatment and behaviour of the majority of the population and the support of the local authorities and representatives of the rural community is a boost.”*

Karima

*“There are some positive factors such as the generosity of the population…”*

Latifa

#### 3.5.3. Empowerment Strategies

##### Soliciting Support from Others

Midwives report that seeking support, advice, and expertise from family, colleagues, provincial health authority officials, and people in the community helps them to feel more adapted and committed to their work.

*“…I often ask the opinion of my colleagues at work and in my profession, and those in charge of the provincial delegation of the Ministry of Health about information that is not communicated to us; we also ask the officials of the rural community who have more information about the needs of the health centre and the population… their support encourages me.”*

Hasna

*“…pointing out the problems that occur at work to the members of the work team and not being left alone to deal with them…”*

Rachida

#### 3.5.4. Complementary/Alternative Activities

##### Activities That Have an Impact on Professional Development

Activities other than professional practice, such as studying for another university degree by distance learning, reading, or languages, empower the midwives and make it practical for them to stay longer in their jobs.

*“My empowerment strategies developed in the work context consist of reading books and magazines, pursuing a university degree in law…”*

Hanane

Working in this context also encourages midwives to empower themselves by maintaining future expectations about resuming their studies, advancing their careers, and participating in continuing education sessions, research projects, congresses, and scientific and cultural meetings where they can share knowledge and experiences with professional colleagues.

*“I aspire to improve my scientific level by continuing my student career and participating in continuing education sessions to follow the latest developments in the profession and scientific and cultural congresses and meetings.”*

Rachida

*“…participation in rare occasions of continuous training that arise to improve the quality of the services provided…”*

Nada

##### Recreational Activities

Despite the limitation of leisure in rural and remote areas of northern Morocco, some recreational activities, such as excursions, are possible. Participating in these activities allows midwives to empower themselves to remain, to think about other aspects not related to their work, and to enjoy the environment where they are working.

*“Participating from time to time in activities for fun and excursions and trips allows us to appreciate these places more…”*

Karima

*“Rarely can we motivate ourselves by organising excursions, but by participating in these modest activities, you go back to work with a bit more motivation and morale.”*

Ikram

## 4. Discussion

We have seen that the midwives lack the basic resources and conditions necessary to care for postpartum women and newborns. Moreover, the working conditions are not conducive to the performance of their role in these environments, which is exacerbated by the personal sacrifices they have to make in order to keep their jobs. This reality leads them to have a negative perception of the quality of work and personal life, and makes them contemplate changing their workplace as soon as this becomes possible. However, some intrinsic and extrinsic factors motivate them to empower themselves and stay in their jobs.

Despite all the contextual, professional, and personal barriers, midwives try to perform their role to the best of their ability with the resources at their disposal. However, they are unable to ensure quality of care as a result of scarce basic resources and poor working conditions, as is the case in other settings [36,37]. Indeed, the Cochrane review [38] of factors influencing the quality of midwifery care delivery in low-income countries concludes that numerous factors, which have also been identified in our study, have a negative impact. These include problems in access to training and lack of supervision, understaffing, excessive workloads, and poor living conditions, as well as poor access to well-equipped and organised health facilities with adequate water, electricity, and transport. Teamwork, trust, collaboration, and communication [38] are achieved according to the informants who participated in the present study, although they recognise a shortage of professionals in the settings in question.

In relation to midwives’ perceptions of their quality of life and work, as well as the factors that support their continuation in difficult work contexts, Cramer (2006) defines nursing practice in rural and remote areas as amorphous [39]. He refers to the changing processes involved, which depend on the context and the means at the nurses’ disposal [39]. This pragmatic perspective of their reality is defined by Wigens (1997) as a rationalisation strategy to minimise feelings of distress associated with conflicting values and beliefs [40]. However, the psychological distress to which midwives are subjected as a result of the mismatch between their knowledge, values, and beliefs and their actions may overwhelm them and lead some of them to leave [41].

Furthermore, our study shows that all five dimensions influencing the attraction and retention of professionals in rural areas in low- and middle-income countries proposed by Lehmann et al. are negatively affected [21]. Our results also concur with those of Belaid et al. [23], namely, that the local environment (e.g., a shortage of basic resources in health care facilities as well as a lack of decent housing); social factors (e.g., isolation, lack of protection, and clients’ mistrust); and working conditions (e.g., lack of training and supervision) negatively affect the appeal of working in rural and remote areas as well as retention rates.

Morocco’s health and social policies do little to promote midwifery in rural and remote areas, leading to a shortage of such staff and an exacerbation of morbidity and mortality rates amongst mothers and their children [11,15]. Government involvement is essential; policy changes are needed to regulate health and social care and provide incentives for health and social care professionals (particularly midwives) in rural and remote areas.

In keeping with the literature review by Lehmann et al. [21], it can be concluded that there is a need to implement intervention packages in order to retain professionals in remote and rural areas. However, developing appropriate strategies for midwives, particularly in the context of low- and middle-income countries, first requires an understanding of the influencing factors that have been addressed in the present study. For example, exchange programmes for midwives and other health professionals from urban to rural areas and vice versa, a have been introduced in other settings [42,43], may not be a welcome strategy in the present case, as working and personal conditions are so harsh.

However, targeting efforts to improve working life, career advancement, and living and family conditions could motivate midwives to accept jobs in rural settings, as the present study and others—such as that conducted in Ghana with student midwives [44]—have demonstrated. It is, therefore, necessary to consider these elements in the development of policies to boost midwifery coverage in rural and remote areas.

In Morocco, intersectoral policies should focus, on the one hand, on providing health care facilities with the necessary infrastructure and material and human resources to ensure decent care for postpartum women and newborns. It is also necessary to improve the road network for the transport of women in labour to referral hospitals, as well as to facilitate the connection of these areas with the most populated ones. Working conditions need to be improved, with a more optimal distribution of the workload as well as reinforced safety, training, and supervision. In addition, it is suggested that shortcomings detected in the present study, such as the provision of housing with decent living conditions, should be addressed, as this could increase the likelihood of midwives accepting rural jobs [45]. At the same time, it is important to foster an optimal working environment as well as recognition of midwives’ work by the authorities and the population. As we have found in our study, in Morocco, there is still a persistent lack of professional recognition of midwives, which is consistent with the widespread gender inequality and low status of women [46,47]. In addition, measures such as salary increases for those working in rural areas might also increase the attractiveness of such positions [45].

Finally, the empowerment of female professionals needs to be strengthened, as this has a direct impact on patient care, patient safety, and the well-being of female professionals [38]. Empowerment contributes positively to improving the environment in which care is provided [48]. As the gaps identified in the present study are overcome, midwives will be better able to develop and engage in actions that empower them, such as daring to ask for help from others or becoming involved in other activities, both for their professional and personal development.

### Limitations

There may have been social desirability bias in the responses of the participants, though we tried to minimise this by ensuring discretion and anonymity. It should be noted that the participants were working in remote and rural areas and were not students (which has been the case in many studies), so the statements they made were not based on expectations, but on reality. The information collected was limited to a small number of people located in several provinces in the north of Morocco, so the results could not be generalised. However, the aim was to gain in-depth knowledge of the reality of the situation, and the findings may reflect the reality in other French-speaking African countries sharing a similar culture.

## 5. Conclusions

Our results show that working and living conditions are important factors in midwives’ perception of their working lives, as well as whether they decide to continue practising their profession in rural and remote environments.

It is important to develop intersectoral policies that focus on providing health care facilities with the necessary materials and human resources to ensure dignified care for women and newborns; to improve the road network for transporting women in labour to referral hospitals; to ensure optimum working conditions, with a fairer distribution of workloads for midwives; to strengthen their safety and professional reputation; to enhance training, supervision, and support; to guarantee decent living conditions; and to provide leisure alternatives.

In addition, health authorities should integrate midwives into work teams, recognise their work (and encourage the same amongst the populations they serve), and support their various empowerment strategies. All of these measures could increase the recruitment and improve the retention rates of midwives in rural and remote areas of northern Morocco, as well as in countries with similar socio-economic and cultural conditions.

## Figures and Tables

**Table 1:** Overview of the sociodemographic characteristics of the sample (n = 15).

Average age (years)			27.86
Average years of employment			3.4
Average number of years of work in current position			2.6
Marital status		Married	8
		Single	7
Educational profile		State registered nurse Grade 1 (DTS 1G)	15
Work modality		Full-time nurse on call	15
Accessibility to workplaces		Difficult	15
Location	Province of Ouzzan	Health centres	2 (4 p)
	Province of Chefchaouen	Health centres	4 (8 p)
	Province of Al-Hoceima	Health centres	2 (3 p)
Distance to the reference hospital (average in km)			60.5
Total number of participants (p)			15

Source: own elaboration. According to the Moroccan Ministry of Health’s classification of hard-to-reach areas in the Tangier-Tetouan-Al Hoceima region (list updated after 2014).

**Table 2:** Description of specific socio-demographic characteristics of the sample (n = 15).

Pseudonymous Name	Age (in Years)	Years of Employment	Years of Employment in Current Position	Educational Profile	Working Hours Full-Time		Marital Status	Number of Children	Distance to Referral Hospital (in km)	Access to Workplace *	Participation	
					Without Shifts	With Shifts					Individual Interview	Focus Group (FG)
Latifa	27	4	2	SRN ^1^		x	Married	1	40	D ^2^	X	GF2
Karima	33	6	4	SRN		x	Single	0	40	D	X	GF2
Nozha	30	2	2	SRN		x	Married	0	40	D	X	
Hasna	32	6	4	SRN		x	Married	1	40	D	X	
Hanane	29	5	3	SRN		x	Married	1	40	D	X	GF2
Rachida	24	5	4	SRN		x	Single	0	45	D	X	GF2
Najoua	28	5	4	SRN		x	Married	1	62	D	X	
Amal	29	3	3	SRN		x	Single	0	137	D	X	
Zohra	25	2	2	SRN		x	Married	0	62	D	X	
Ikram	27	2	2	SRN		x	Single	0	62	D	X	GF1
Naziha	35	4	4	SRN		x	Single	0	57	D	X	GF1
Laila	25	1	1	SRN		x	Single	0	57	D	X	GF1
Hind	25	2	2	SRN		x	Married	0	53	D	X	GF3
Nada	24	1	1	SRN		x	Single	0	53	D	X	GF3
Ibtissam	25	3	1	SRN		x	Married	1	120	D	X	GF3

^1^ SRN: State registered nurse. ^2^ D: Difficult. * According to the Moroccan Ministry of Health’s classification of hard-to-reach areas in the Tangier-Tetouan-Al Hoceima region (list updated after 2014).

## Data Availability

Not applicable.
